# Prognostic Implications of Preoperative hs-cTnT in Elective Coronary Artery Bypass Grafting

**DOI:** 10.1016/j.jacadv.2025.102180

**Published:** 2025-09-23

**Authors:** Leo Pölzl, Joseph Kletzer, Ronja Lohmann, Christian Sutter, Maria Ioannou-Nikolaidou, Clemens Engler, Michael Graber, Felix Nägele, Jakob Hirsch, Samuel Heuts, Martin Czerny, Sebastian J. Reinstadler, Johannes Holfeld, Michael Grimm, Albi Fagu, Maximilian Kreibich, Tau Hartikainen, Nikolaos Bonaros, Tim Berger, Can Gollmann-Tepeköylü

**Affiliations:** a Department of Cardiac Surgery, Medical University of Innsbruck, Tyrol, Innsbruck, Austria; b Department of Cardiovascular Surgery, University Heart Center Freiburg-Bad Krozingen, University Medical Center Freiburg, Freiburg, Germany; c Faculty of Medicine, University of Freiburg, Freiburg, Germany; d Faculty of Medicine, Paracelsus Medical University, Salzburg, Austria; e Cardio-Thoracic Surgery Department, Maastricht University Medical Centre, Maastricht, the Netherlands; f Department of Internal Medicine III, Cardiology and Angiology, Medical University of Innsbruck, Innsbruck, Austria; g Department of Cardiovascular Surgery, German Heart Center Munich, School of Medicine & Health, Technical University of Munich, Munich, Germany; h Department of Cardiology and Angiology, University Heart Center Freiburg-Bad Krozingen, University Medical Center Freiburg, Freiburg, Germany

**Keywords:** biomarkers, CABG, cardiac surgery, cardiac troponin, risk stratification

## Abstract

**Background:**

Elevated preoperative hs-cTnT may reflect underlying myocardial vulnerability, potentially influencing surgical timing and perioperative strategies in patients undergoing elective coronary artery bypass grafting (CABG).

**Objectives:**

This study investigates the association between preoperative hs-cTnT levels, perioperative outcomes, and long-term mortality, aiming to improve risk stratification and guide clinical decision-making.

**Methods:**

This retrospective study analyzed a consecutive series of 5,450 patients undergoing CABG at 2 tertiary centers between 2010 and 2023. Patients were categorized into 3 groups based on preoperative hs-cTnT levels: 1) nonelevated (<1x upper reference limit [URL]); 2) mildly elevated (1–3x URL); and 3) significantly elevated (>3x URL). A propensity score weighting method was performed before evaluating the association of hs-cTnT with perioperative outcomes, 30-day mortality and 5-year mortality.

**Results:**

Among elective CABG patients, 26.6% had hs-cTnT levels >1x URL, and 12.4% had levels >3x URL. Patients with significantly elevated hs-cTnT (>3x URL) demonstrated increased risks of extracorporeal membrane oxygenation use (HR: 2.96 [95% CI: 1.81-4.84]), hemofiltration (HR: 2.99 [95% CI: 2.27-3.94]), and 5-year mortality (HR: 1.55 [95% CI: 1.28-1.86]) (all *P* < 0.001). Even mild elevations (1–3x URL) were linked to higher rates of hemofiltration (HR: 2.25 [1.75-2.90]; *P* < 0.001), extracorporeal membrane oxygenation use (HR: 1.65 [95% CI: 1.01-2.69]; *P* = 0.046), and 5-year mortality (HR: 1.37 [95% CI: 1.14-1.34]; *P* < 0.001).

**Conclusions:**

Preoperative hs-cTnT is an independent predictor of adverse outcomes in elective CABG. Integrating hs-cTnT into routine preoperative assessment could identify high-risk patients, optimize surgical timing, and determine whether patients may benefit more from CABG or percutaneous coronary intervention, ultimately improving clinical outcomes.

Coronary artery bypass grafting (CABG) remains a cornerstone in the treatment of complex coronary artery disease (CAD), serving as the gold standard for surgical revascularization with over 400,000 procedures performed annually in the United States.^1^ It is the preferred treatment for patients with complex or multivessel CAD, significantly improving survival and quality of life. Accurate preoperative risk stratification is critical for identifying high-risk patients and optimize outcomes.^2^

Cardiac troponin, a highly specific biomarker for myocardial injury, has transformed the assessment and diagnosis of ischemic heart disease. Even in the absence of clinically apparent myocardial infarction, elevated cardiac troponin levels have been closely associated with poor cardiovascular outcomes, including increased mortality.^3^ Recent advancements in high-sensitivity assays (hs-cTn) enable the detection of even minor myocardial injury, offering new avenues for risk stratification and prognostic evaluation.^4^ Elevated cardiac troponin levels have demonstrated prognostic significance not only in acute coronary syndrome and percutaneous coronary interventions but also in cardiac surgery, where perioperative myocardial injury remains a serious complication.^5^^,^^6^

Substantial evidence links postoperative troponin elevations to adverse outcomes after cardiac surgery.^4^^,^^6^ Data on preoperative hs-cTnT levels in the context of CABG focus on emergency settings or noncardiac surgeries. Early findings suggest that preoperative troponin elevations may indicate underlying subclinical myocardial injury and serve as independent predictors of postoperative mortality and morbidity.^7^^,^^8^ Despite these insights, a significant knowledge gap remains regarding the prognostic value of preoperative hs-cTn in patients undergoing elective CABG.^9^

In this study, we aim to bridge this gap by analyzing: 1) the incidence of preoperatively elevated hs-cTnT levels; and 2) their association with clinical outcomes following elective CABG.

## Methods

### Study population, data collection, and ethical approval

Data from 5,739 consecutive patients undergoing CABG between 2010 and 2023 from 2 tertiary centers were analyzed retrospectively. Two hundred eighty-nine patients were excluded due to missing cardiac biomarkers or missing data on the primary endpoint. The final study cohort consisted of 5,450 patients (Innsbruck, Austria, n = 3,033; Freiburg, Germany n = 2,417). The indication for CABG was determined through an interdisciplinary decision-making process involving cardiologists and cardiac surgeons. Surgical strategies, including the choice and number of grafts, were at the discretion of the operating surgeon.

High-sensitivity cardiac troponin T (hs-cTnT) levels were routinely measured preoperatively (the day before surgery) and serially after surgery using the Elecsys Troponin T-high-sensitivity assay (Roche Diagnostics) (upper reference limit [URL] = 14 ng/L) in both centers. Approximately 50% of patients had values exceeding this threshold, with elevations ranging up to 3,023 ng/L, highlighting the need for further stratification. To identify patients with markedly elevated hs-cTnT, we applied a secondary threshold of 3× URL (42 ng/L). Accordingly, patients were categorized into 3 groups: a) normal (<1× URL), b) mildly elevated (1-3× URL), and c) significantly elevated (>3× URL) preoperative hs-cTnT levels.

Follow-up data were collected through chart reviews, telephone interviews, and survival data from governmental authorities, ensuring 100% follow-up completion. The study was conducted in accordance with the Declaration of Helsinki. Ethical approval for the use of anonymized patient data without individual consent was obtained from the Institutional Review Boards of Innsbruck Medical University (UN 1009/2024) and the University of Freiburg (24-1248-S1-retro). To ensure robustness of the results, sensitivity analysis including only elective patients and patients without impairment of renal function were included.

### Study endpoints and statistical analysis

The primary outcome of this study was all-cause mortality within 5 years after CABG. Secondary outcomes included 30-day mortality, duration of intensive care unit (ICU) stay, and the need for extracorporeal membrane oxygenation (ECMO) or ultrafiltration. Continuous variables are reported as median (IQR), while categorical variables are presented as frequencies and percentages. Group comparisons were performed using the Kruskal-Wallis rank sum test for continuous variables, and Pearson's chi-squared test or analysis of variance for categorical variables, as appropriate.

We utilized a propensity score weighing method as proposed in the Toolkit for Weighing and Analysis of Nonequivalent Groups (twang) package for R following instructions layed out in the vignettes accompanying the package.^10^ Firstly, to analyze average treatment effect, propensity scores were estimated using gradient boosted models decomposed into several functions, as per the mnps function of the twang package. Factors used for weighing were determined by imbalances found in the summary statistic (Table 1). Factors used were age, sex, body mass index, hypertension, diabetes mellitus, dyslipidemia, peripheral artery disease, dialysis, chronic obstructive pulmonary disease, smoking status, history of stroke, previous myocardial infarction, previous percutaneous intervention, as well as NYHA score. Hyperparameters such as the “stop method,” or number of gradient boosting iterations were chosen based on serial experimentation and comparison of achieved balance (Supplemental Figure 1). In this case, we used the Kolmogorov-Smirnov statistic summarized across the pretreatment variable by the mean as a stopping method, 5,000 iterations of gradient boosting, a learning rate of 0.01, a tree depth of 3, and a minimum number of observations in the terminal tree nodes of 10. Finalized balance was assessed using absolute standard difference and visualized in Supplemental Figure 2. All subsequent analyses utilized the weights yielded from these steps.

**Table 1:** Baseline Characteristics

	Overall (N = 5,450)	URL of hs-cTnT			*P* Value
		Normal Range (n = 2,856)	14-42 ng/l hs-cTnT (n = 1,423)	≥42 ng/dL hs-cTnT (n = 1,171)	
Female	866 (16%)	472 (17%)	188 (13%)	206 (18%)	0.004
Age (y)	68.00 (60.62, 74.00)	66.00 (59.06, 72.14)	70.68 (63.74, 76.14)	69.00 (61.00, 75.00)	<0.001
Body mass index (kg/m^2^)	27.00 (24.98, 30.00)	27.00 (25.00, 30.00)	27.40 (25.00, 30.11)	27.10 (24.69, 30.20)	0.12
History of hypertension	4,852 (89%)	2,517 (88%)	1,289 (91%)	1,046 (89%)	0.032
Diabetes mellitus	1,885 (35%)	860 (30%)	593 (42%)	432 (37%)	<0.001
Insulin-dependent diabetes mellitus	569 (11%)	200 (7.7%)	210 (16%)	159 (14%)	<0.001
Dyslipidemia	4,757 (87%)	2,557 (90%)	1,236 (87%)	964 (82%)	<0.001
Peripheral artery disease	662 (14%)	302 (12%)	217 (18%)	143 (14%)	<0.001
Preoperative on dialysis	70 (1.5%)	8 (0.3%)	16 (1.3%)	46 (4.5%)	<0.001
Preoperative GFR <30 mL/min	304 (5.9%)	110 (4.1%)	71 (5.4%)	123 (11%)	<0.001
History of COPD	506 (9.3%)	230 (8.1%)	142 (10.0%)	134 (11%)	0.002
History of smoking	2,311 (45%)	1,250 (45%)	585 (43%)	476 (44%)	0.40
History of stroke	320 (5.9%)	128 (4.5%)	110 (7.7%)	82 (7.0%)	<0.001
History of myocardial infarction	2,327 (43%)	885 (31%)	601 (42%)	841 (72%)	<0.001
Previous PCI	851 (26%)	511 (27%)	211 (25%)	129 (22%)	0.070
NYHA functional class					<0.001
I	641 (14%)	379 (16%)	138 (11%)	124 (12%)	
II	2,206 (47%)	1,265 (52%)	578 (47%)	363 (35%)	
III	1,681 (36%)	745 (31%)	492 (40%)	444 (43%)	
IV	158 (3.4%)	23 (1.0%)	31 (2.5%)	104 (10%)	
Preoperative LVEF <30%	155 (2.9%)	32 (1.1%)	52 (3.7%)	71 (6.1%)	<0.001
Preoperative hs-cTnT(ng/l)	13.40 (8.00, 32.00)	8.00 (5.90, 10.80)	21.00 (17.00, 27.30)	181.90 (72.95, 512.50)	<0.001

Values are n (%) or median (IQR).

COPD = chronic obstructive pulmonary disease; GFR = glomerular filtration rate; LVEF = left ventricular ejection fraction; PCI = percutaneous coronary intervention; URL = upper reference limit.

Time zero was defined as the time of the surgical procedure, with follow-up censored at 5 years, resulting in a median follow-up time of 5.00 (2.27-5.00) years. Kaplan-Meier survival curves were generated for all-cause mortality, and survival distributions were compared using log-rank tests. To evaluate the association between preoperative hs-cTnT levels and outcomes, a multivariable mixed-effects Cox regression analysis was performed. The proportional hazards assumption was assessed using Schoenfeld residual plots. European System for Cardiac Operative Risk Evaluation (EuroSCORE) II was included as a covariate in all regression models to adjust for preoperative comorbidities. Results are reported as HRs with 95% CIs. A 2-sided *P* value <0.05 was considered statistically significant. All statistical analyses were conducted using R software (Version 4.3.3, R Core Team, 2024).

## Results

### Patient cohort and surgical characteristics

Among the 5,450 patients included in the study, 2,856 (52.4%) had preoperative hs-cTnT levels within the normal range, 1,423 (26.1%) had mildly elevated levels (14-42 ng/L), and 1,171 (21.5%) had significantly elevated levels (≥42 ng/L) (Supplemental Figure 3). Patients with elevated hs-cTnT levels were older, with median ages of 70.7 years in the 14 to 42 ng/L group and 69.0 years in the ≥42 ng/L group, compared to 66.0 years in the normal range group (*P* < 0.001). The proportion of female patients was significantly lower in the 14 to 42 ng/L group (13%) compared to the normal range (17%) and the ≥42 ng/L group (18%) (*P* = 0.004).

Comorbidities were notably more frequent in patients with elevated hs-cTnT. The prevalence of diabetes mellitus increased progressively from 30% in the normal range group to 42% in the 14 to 42 ng/L group and 37% in the ≥42 ng/L group (*P* < 0.001). Similarly, insulin-dependent diabetes mellitus was significantly more common in the 14 to 42 ng/L group (16%) and the ≥42 ng/L group (14%) compared to the normal range group (7.7%) (*P* < 0.001). Renal dysfunction, as indicated by preoperative glomerular filtration rate <30 mL/min, was substantially more prevalent in the ≥42 ng/L group (11%) compared to the normal range group (4.1%) (*P* < 0.001).

NYHA functional class worsened with increasing hs-cTnT levels. In the normal range group, 52% of patients were classified as NYHA functional class II, whereas only 35% in the ≥42 ng/L group fell into this category. Conversely, higher proportions of NYHA functional class III (43%) and class 4 (10%) were observed in the ≥42 ng/L group (*P* < 0.001). Left ventricular function was significantly impaired in patients with elevated hs-cTnT levels. A preoperative left ventricular ejection fraction <30% was found in 6.1% of patients in the ≥42 ng/L group compared to only 1.1% in the normal range group (*P* < 0.001) (all Table 1).

EuroSCORE II increased progressively with higher hs-cTnT levels, with median values of 1.53% in the normal range group, 2.40% in the 14 to 42 ng/L group, and 3.95% in the ≥42 ng/L group (*P* < 0.001). Surgical urgency differed significantly across the groups. Elective procedures were most frequent in the normal range group (85%), declining to 75% in the 14 to 42 ng/L group and 41% in the ≥42 ng/L group. In contrast, urgent and emergency/salvage procedures were substantially higher in the elevated troponin groups. In the ≥42 ng/L group, 24% of surgeries were classified as urgent, while 35% were emergency or salvage procedures (*P* < 0.001).

Intraoperative variables also varied with troponin levels. Median cross-clamp time increased from 68 minutes in the normal range group to 72 minutes in the 14 to 42 ng/L group (*P* < 0.001). Cardiopulmonary bypass time followed a similar trend, with median times of 99 minutes in the normal range group and 102 minutes in the elevated troponin groups (*P* < 0.001).

### Preoperative hs-cTnT is associated with outcome after CABG

Postoperative outcomes were markedly worse in patients with elevated hs-cTnT. The need for ECMO increased progressively, with 3.8% of patients in the ≥42 ng/L group requiring ECMO compared to 0.6% in the normal range group (*P* < 0.001). ICU length of stay also increased significantly, with patients in the ≥42 ng/L group having a median ICU stay of 1 day (IQR: 0-4) compared to 0 days (IQR: 0-1) in the normal range group (*P* < 0.001). Postoperative ultrafiltration was also more frequent in patients with elevated hs-cTnT, occurring in 7.5% of patients in the 14 to 42 ng/L group and 10% in the ≥42 ng/L group, compared to 2.2% in the normal range group (*P* < 0.001).

Postoperative hs-cTnT levels reflected the severity of myocardial injury, with median peak concentrations of 1,045.5 ng/L in the ≥42 ng/L group compared to 807.5 ng/L in the normal range group (*P* < 0.001). Mortality at 30 days followed a similar trend, increasing to 3.7% in the ≥42 ng/L group compared to 0.9% in the normal range group (*P* < 0.001) (all Table 2).

**Table 2:** Surgical Characteristics and Outcome

		URL of hs-cTnT			
	Overall N = 5,450	Normal Range (n = 2,856)	14-42 ng/l hs-cTnT (n = 1,423)	≥42 ng/dL hs-cTnT (n = 1,171)	*P* Value
EuroSCORE II (%)	2.28 (2.78)	1.53 (1.52)	2.40 (2.06)	3.95 (4.60)	<0.001
Urgency					<0.001
Elective	3,673 (73%)	2,240 (85%)	979 (75%)	454 (41%)	
Urgent	389 (7.7%)	60 (2.3%)	63 (4.8%)	266 (24%)	
Emergency/Salvage	993 (20%)	339 (13%)	266 (20%)	388 (35%)	
Cross-clamp time (min)	70 (56, 85)	68 (55, 83)	72 (58, 86)	70 (58, 86)	<0.001
Cardiopulmonary bypass time (min)	101 (83, 123)	99 (82, 121)	102 (85, 124)	102 (83, 126)	<0.001
Outcome					
ECMO	75 (1.5%)	15 (0.6%)	18 (1.4%)	42 (3.8%)	<0.001
Days in the ICU	0 (0, 1)	0 (0, 1)	0 (0, 2)	1 (0, 4)	<0.001
Postoperative ultrafiltration	269 (5.3%)	58 (2.2%)	99 (7.5%)	112 (10%)	<0.001
Postoperative hs-cTnT (ng/L)	848.45 (506.00, 1,407.50)	807.45 (478.50, 1,284.50)	821.05 (494.10, 1,337.75)	1,045.50 (591.50, 1,795.00)	<0.001
Died after 30 days	87 (1.6%)	25 (0.9%)	19 (1.3%)	43 (3.7%)	<0.001

Values are n (%) or median (IQR).

ECMO = extracorporeal membrane oxygenation; ICU = intensive care unit; other abbreviation as in Table 1.

**Figure 1:**
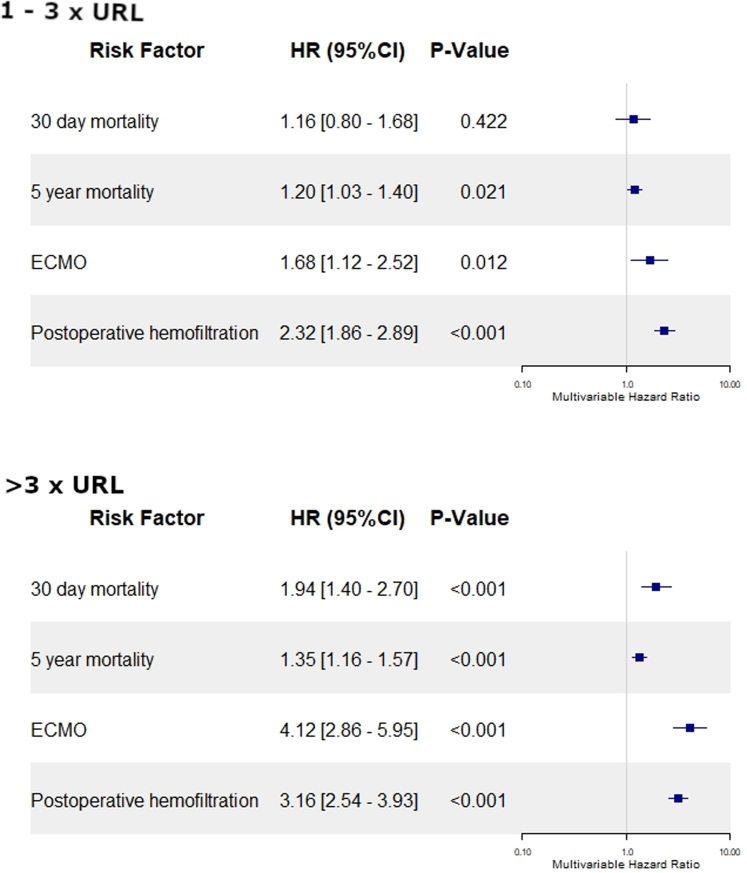
**Preoperative hs-cTnT Is an Independent Risk Factor for the Outcome After CABG** Patients were grouped based on their preoperative hs-cTnT values and a pseudo randomization using a propensity score weighing method was performed. patients with preoperative hs-cTnT 1x – 3x URL, and in >3x URL were compared to <1x URL. Models were adjusted for the EuroSCORE II. ECMO = extracorporeal membrane oxygenation; URL = upper reference limit; CABG = coronary artery bypass grafting.

**Figure 2:**
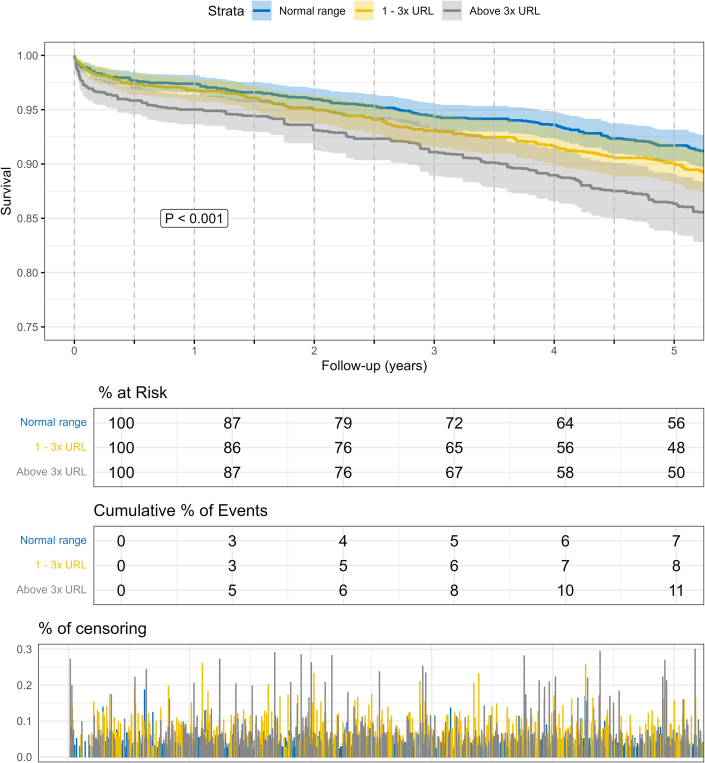
**5-Year Mortality is Dependent on Preoperative hs-cTnT** Patients were grouped based on their preoperative hs-cTnT values and a pseudo randomization using a propensity score weighing method was performed (<1x URL = blue; 1-3x URL = yellow; > 3xURL); abbreviation as in Figure 1.

To elucidate the effect of the preoperative hs-cTnT, a pseudo randomization propensity score weighing method was performed. Within the multivariable regression model, already slightly increased preoperative hs-cTnT levels (1-3× URL) were associated with postoperative ECMO (HR: 1.678 [95% CI: 1.116-2.523]; *P*: 0.012) and ultrafiltration (HR: 2.316 [95% CI: 1.856-2.890], *P* < 0.001) (Figure 1). Kaplan-Meier curves revealed a significantly increased 5-year mortality in patients with preoperative hs-cTnT >1× URL (*P* < 0.001) (Figure 2). These findings were supported by results of the multivariable model (1-3× URL: HR: 1.200 [95% CI: 1.028-1.400]; *P* = 0.021) (Figure 1).

Within the multivariable regression model, adjusted for all preoperative characteristics, preoperative hs-cTnT levels >3 URL were associated with postoperative ECMO (HR: 4.123 [95% CI: 2.860-5.945]; *P* < 0.001) and ultrafiltration (HR: 3.157 [95% CI: 2.538-3.927], *P* < 0.001) (Figure 1). Within Kaplan-Meier curves and the multivariable regression model, an increased risk for 30-day and 5-year mortality was observed (30-day: HR: 1.943 [95% CI: 1.398-2.699]; 5-year: HR: 1.346 [95% CI: 1.157-1.566]; both *P* < 0.001) (Figure 2, Supplemental Figure 4).

The same applies to the comparison of the nonweighted groups (Supplemental Figure 5 to 7).

To enable risk stratification after cardiac surgery for the individual patient, we performed regression analyses for different EuroSCORE values and preparative hs-cTnT levels.

Table 3 shows the modification of the expected 30-day mortality (EuroSCORE II) according to the preoperative hs-cTnT levels for the individual patient.

**Table 3:** Association of Preoperative hs-cTnT and EuroSCORE II With the 30-Day Mortality

	hs-cTnT		
	Nonelevated *(<1x URL)*	Mildly Elevated *(1-3x URL)*	Significantly Elevated *(>3x URL)*
EuroSCORE II, n (%)			
<1%	1,127 (27.7%)	175 (4.3%)	97 (2.4%)
1%-2%	992 (24.4%)	469 (11.5%)	208 (5.1%)
>2%	404 (9.9%)	384 (9.4%)	212 (5.2%)
EuroSCORE II, OR (95% CI)			
<1%	Ref.	2.153 (0.223-20.817	0
1%-2%	3.43 (0.926-12.706)	3.223 (0.719-14.456)	5.483 (1.099-27.354)
>2%	8.537 (2.300-31.691)	6.957 (1.790-27.038)	14.693 (3.86655.847)
EuroSCORE II, *P* value			
<1%	Ref.	0.508	0.997
1%-2%	0.065	0.126	0.038
>2%	0.001	0.005	<0.001

Elective patients were grouped based on their preoperative hs-cTnT values and the EuroSCORE II levels. Patients with preoperative hs-cTnT 1x – 3x URL and an EuroSCORE II <1% were used as reference.

Abbreviation as in Table 1.

### Preoperative hs-cTnT LEVELS are correlated with outcomes following elective CABG

**Figure 3:**
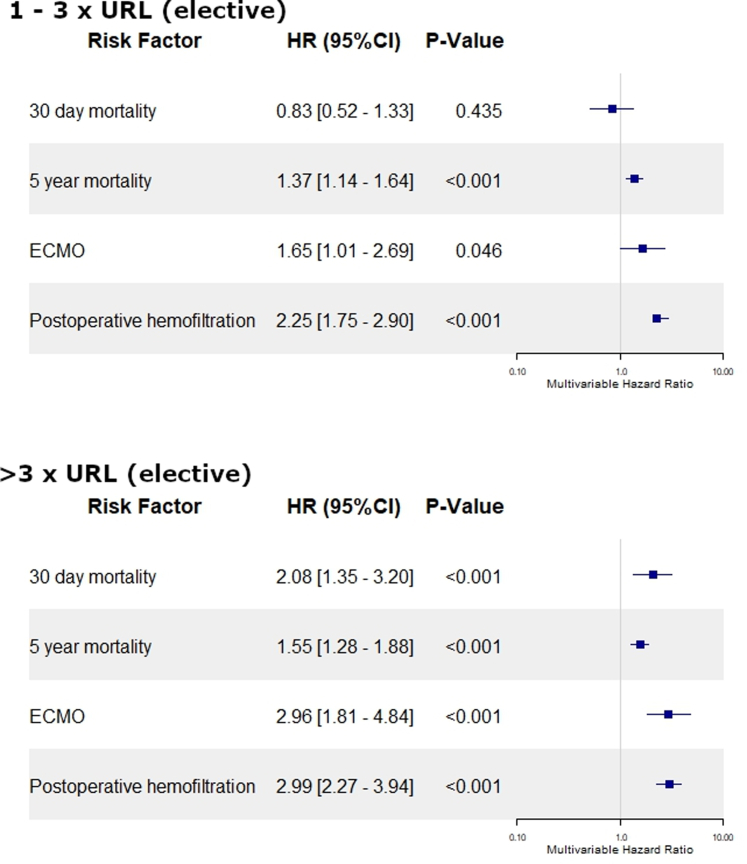
**Preoperative hs-cTnT Is an Independent Risk Factor for the Outcome After Elective CABG** Only elective patients were analyzed and grouped based on their preoperative hs-cTnT values. A pseudo randomization using a propensity score weighing method was performed. Patients with preoperative hs-cTnT in 1x – 3x URL, and >3x URL were compared to <1x URL. Model was adjusted for the EuroSCORE II. Abbreviations as in Figure 1.

**Figure 4:**
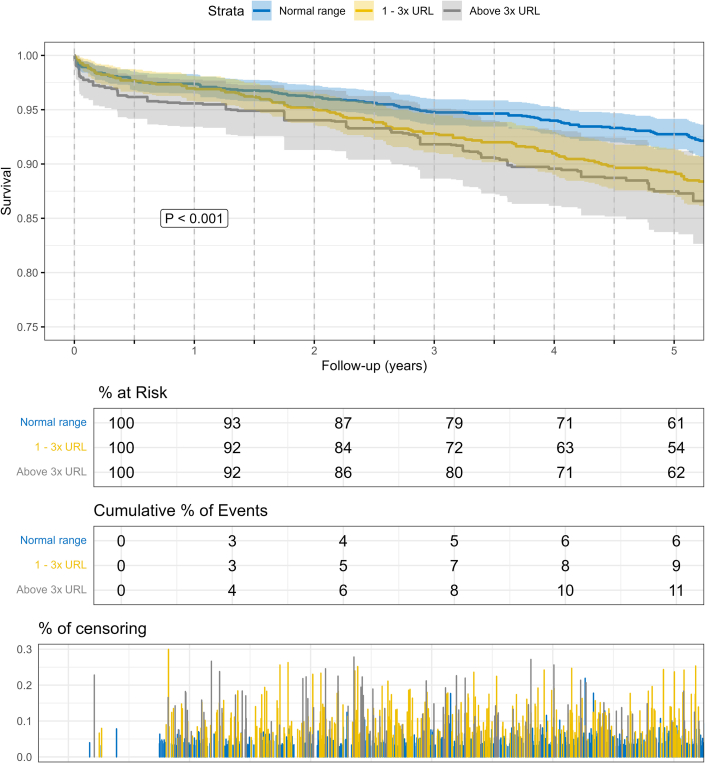
**5-Year Mortality Is Dependent on Preoperative hs-cTnT in Elective Patients** Only elective patients were analyzed and grouped based on their preoperative hs-cTnT values. A pseudo randomization using a propensity score weighing method was performed. (<1x URL = blue; 1-3x URL = yellow; > 3xURL); abbreviation as in Figure 1.

In a next step, only elective patients were analyzed. Elective surgery was performed in 73% of patients. Nevertheless, 26.65% had preoperative hs-cTnT levels >1× URL and 12.36% >3x URL. Increased preoperative hs-cTnT levels were associated with increased rate of ultrafiltration (1-3× URL: HR: 2.252 [95% CI: 1.747-2.903]; >3x URL: HR: 2.988 [95% CI: 2.265-3.942]; both *P* <0.001), increased ECMO rate (1-3× URL: HR: 1.647 [95% CI: 1.008-2.691], *P* = 0.046; >3× URL: HR: 2.958 [95% CI: 1.809-4.836]; *P* <0.001), and 5-year mortality (1-3× URL: HR: 1.367 [95% CI: 1.142-1.636]; >3× URL: HR: 1.551 [95% CI: 1.284-1.875]; both *P* < 0.001). hs-cTnT levels >3× URL were associated with increased 30-day mortality (HR: 2.077 [95% CI: 1.348-3.199]; *P* < 0.001) (all Figure 3). These findings were supported by Kaplan-Meier curves demonstrating increased 30-day and 5-year mortality rates in patients with preoperative hs-cTnT >1× URL and >3x URL, respectively (Figure 4, Supplemental Figure 8). The same applies to the comparison of the nonweighted groups (Supplemental Figures 9 to 11). hs-cTnT values depend to a certain degree on the renal function. Therefore, for sensitivity analysis, we repeated the analysis in patients with a creatinine clearance value <0.9 mg/dL in women and <1.2 mg/dL in men. The Kaplan-Meier curve revealed increased 5-year mortality in patients with increased preoperative hs-cTnT values (Supplemental Figures 12 and 13).

## Discussion

This retrospective propensity score-weighted study of 5,450 patients undergoing CABG highlights the significant prognostic value of preoperative hs-cTnT levels (Central Illustration). Elevated hs-cTnT (>3x URL) was independently associated with higher rates of ECMO use, hemofiltration, 30-day, and 5-year mortality. Even modest elevations (1–3x URL) were predictive of adverse outcomes, including increased hemofiltration and 5-year mortality. These associations remained robust in elective patients and sensitivity analyses restricted to those with preserved renal function, suggesting that elevated hs-cTnT reflects underlying myocardial injury rather than impaired clearance. These findings underscore the importance of hs-cTnT as a biomarker for risk stratification and decision-making in CABG patients.

**Central Illustration:**
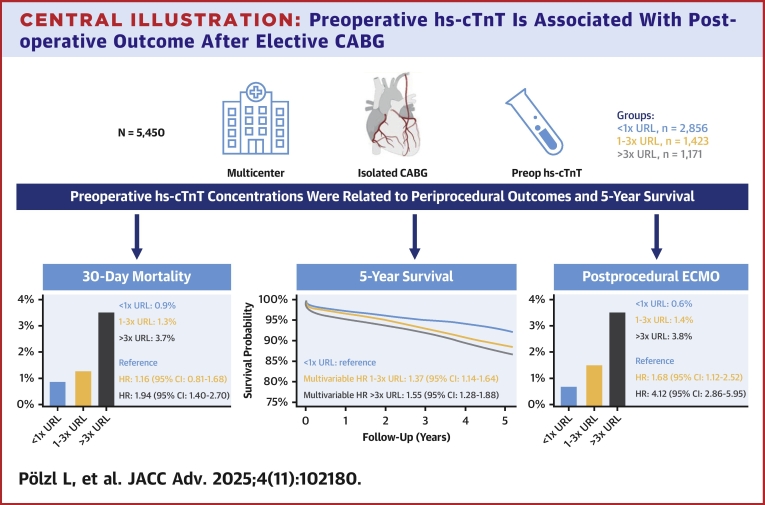
**Preoperative hs-cTnT Is Associated With Postoperative Outcome After Elective CABG** In this retrospective study, 5,450 patients undergoing CABG at 2 tertiary centers were analyzed. Patients were stratified into 3 groups based on preoperative hs-cTnT levels: (a) nonelevated (<1x URL), (b) mildly elevated (1–3x URL), and (c) significantly elevated (>3x URL). Preoperative hs-cTnT levels were an independent predictor for the short- and long-term outcome after CABG in a propensity score weighted model. Already mildly elevated hs-cTnT levels were associated with an increased long-term mortality. Abbreviations as in Figure 1.

CABG, the recommended treatment for patients with complex or multivessel CAD, is the most frequently performed cardiac surgery.^1^ Despite its clinical benefit, CABG carries considerable perioperative risks, emphasizing the need for comprehensive preoperative risk assessment. While cardiac troponins have been validated as risk stratification tools in noncardiac surgery,^7^^,^^8^^,^^11^ data on their role in elective CABG remain sparse.^12^^,^^13^ Our study bridges this knowledge gap by demonstrating the prognostic importance of preoperative hs-cTnT levels and their correlation with both short- and long-term adverse outcomes in a large multicenter cohort.

The observed association between elevated preoperative hs-cTnT and adverse outcomes is consistent with previous studies in patients undergoing CABG for acute myocardial infarction.^9^ Elevated hs-cTnT likely reflects ongoing myocardial injury, ischemia, or a reduced overall cardiac reserve. Patients with higher hs-cTnT levels exhibited a higher prevalence of comorbidities, including diabetes, renal dysfunction, and impaired cardiac function, and poorer functional status based on NYHA classification. These findings suggest that elevated hs-cTnT serves as a surrogate marker for the cumulative burden of cardiovascular risk factors, which, in turn, contribute to worse postoperative outcomes. However, even after propensity score weighting, elevated preoperative hs-cTnT levels remained associated with impaired perioperative outcomes and survival.

In elective patients, hs-cTnT levels >3x URL were independently associated with increased rates of ECMO, hemofiltration, and long-term mortality, irrespective of comorbidities captured by the EuroSCORE II. Importantly, even modest hs-cTnT elevations (1–3x URL) were predictive of adverse outcomes, particularly higher rates of hemofiltration and 5-year mortality. These findings are clinically relevant as minor hs-cTnT elevations are often overlooked in routine practice. Given the strong and independent predictive value of hs-cTnT, integrating its measurement into preoperative risk assessment could enhance patient stratification and guide perioperative management strategies.

We initially hypothesized that elevated hs-cTnT levels might at least partly result from reduced renal clearance, as renal dysfunction is a known confounder of elevated troponin levels.^14^, ^15^, ^16^, ^17^ However, sensitivity analyses restricted to patients with preserved renal function revealed persistent associations between elevated hs-cTnT and impaired outcomes. These findings suggest that hs-cTnT elevation primarily reflects myocardial injury rather than impaired troponin clearance.

The pathophysiological mechanisms underlying elevated hs-cTnT levels likely involve ongoing “silent” myocardial ischemia or a generally compromised coronary status. Silent ischemia, characterized by ischemic episodes without overt clinical symptoms, may occur in patients with severe multivessel CAD or microvascular dysfunction. In patients with complex CAD, increased preoperative hs-cTnT levels were observed earlier.^18^

Conditions such as myocardial infarction with nonobstructive coronary arteries further exemplify how subtle ischemic damage can lead to elevated cardiac biomarkers.^19^^,^^20^

In elective patients with significantly elevated hs-cTnT (>3x URL), further preoperative work-up may be warranted, including detailed clinical examination, serial hs-cTnT measurements, and electrocardiography monitoring, to identify potential dynamic changes indicative of acute coronary syndrome.^21^ Detecting dynamic changes could differentiate between chronic myocardial injury and acute ischemic events, informing decisions regarding the timing of surgery, and potentially postponement—or expedition—of the procedure.

The optimal management of patients with elevated preoperative hs-cTnT remains an area of debate. One possible strategy involves postponing surgery to allow resolution of silent ischemia, thereby reducing the risk of perioperative myocardial injury. However, delaying surgery could also exacerbate ischemic injury, particularly in patients with ongoing myocardial compromise. Notably, we observed higher postoperative hs-cTnT levels in patients with elevated preoperative levels, suggesting greater vulnerability to perioperative myocardial injury. This raises the possibility that early surgical intervention may mitigate the progression of myocardial damage. Potential clinical implications, as the impact of surgical timing on clinical outcomes in patients with elevated preoperative hs-cTnT levels, have to be tested in future prospective studies.

Our findings support the use of preoperative hs-cTnT levels as a robust risk stratification tool for patients undergoing CABG. By reflecting the global health status of patients, including cardiac reserve and comorbidities, hs-cTnT can identify individuals at higher risk of adverse outcomes. Additionally, hs-cTnT levels may inform the choice of revascularization strategy, particularly in patients with chronic coronary syndromes. While CABG is the preferred option for multivessel disease, elevated hs-cTnT levels might indicate higher perioperative risks, prompting consideration of percutaneous coronary intervention as an alternative. Due to the exploratory nature of this study, further studies are needed to explore thresholds of hs-cTnT that may guide the decision-making process between CABG and percutaneous coronary intervention.

### Study Limitations

The strengths of our study include its large cohort size, detailed preoperative biomarker analysis, and robust outcome measures. However, several limitations warrant consideration including all the limitations of a retrospective study. We performed a propensity score weighted analysis, which can only account for observed confounders, potentially leaving the results biased by unmeasured or unknown variables. While sensitivity analyses accounted for renal function, other unmeasured confounders may have influenced the results.

Future research should focus on prospective validation of hs-cTnT as a preoperative risk stratification tool in CABG patients. Randomized trials evaluating the optimal management strategy for patients with elevated hs-cTnT, including the timing of surgery and preoperative interventions, are needed. Additionally, studies exploring thresholds for integrating hs-cTnT into decision-making algorithms for percutaneous coronary intervention vs CABG would provide further clinical insight. Postoperative troponin release is associated with the 30-day mortality after CABG. Studies investigating the relationship between preoperative and postoperative troponin levels still need to be conducted. An influence of this dynamic on the postoperative outcomes cannot be ruled out.

## Conclusions

In summary, preoperative hs-cTnT levels, even at modest elevations, are independently associated with impaired short- and long-term outcomes after elective CABG. The high sensitivity of modern cardiac biomarker assays enables the detection of subtle myocardial injury, making hs-cTnT a valuable tool for preoperative risk stratification. Routine assessment of hs-cTnT in addition to established risk stratification tools could enhance patient evaluation, inform clinical decision-making, and identify high-risk individuals who may benefit from tailored management strategies. Further research is warranted to optimize perioperative care and improve outcomes in this vulnerable patient population.

Perspectives **COMPETENCY IN PATIENT CARE**

Elevated preoperative hs-cTnT levels have been linked to myocardial vulnerability, but the impact on perioperative outcomes and long-term mortality in elective CABG patients remains unclear. This study demonstrates that even mildly elevated hs-cTnT levels are associated with increased risks of ECMO use, hemofiltration, and 5-year mortality in patients undergoing elective CABG.

**TRANSLATIONAL OUTLOOK**

Future studies should explore strategies to optimize patients with elevated hs-cTnT levels, including individualized perioperative management, and assess the efficacy of cardioprotective interventions to improve outcomes in this high-risk group.

## Funding support and author disclosures

The authors have reported that they have no relationships relevant to the contents of this paper to disclose.
