# Supplementary material for: Prognostic Implications of Preoperative hs-cTnT in Elective Coronary Artery Bypass Grafting
Source: JACC Adv. 2025 Sep 23;4(11):102180. doi: 10.1016/j.jacadv.2025.102180 (PMC12495324; doi:10.1016/j.jacadv.2025.102180)
Supplement: Supplemental material [file mmc1.docx]

**Supplemental Figure 1:** Propensity score weighing - Estimated balance


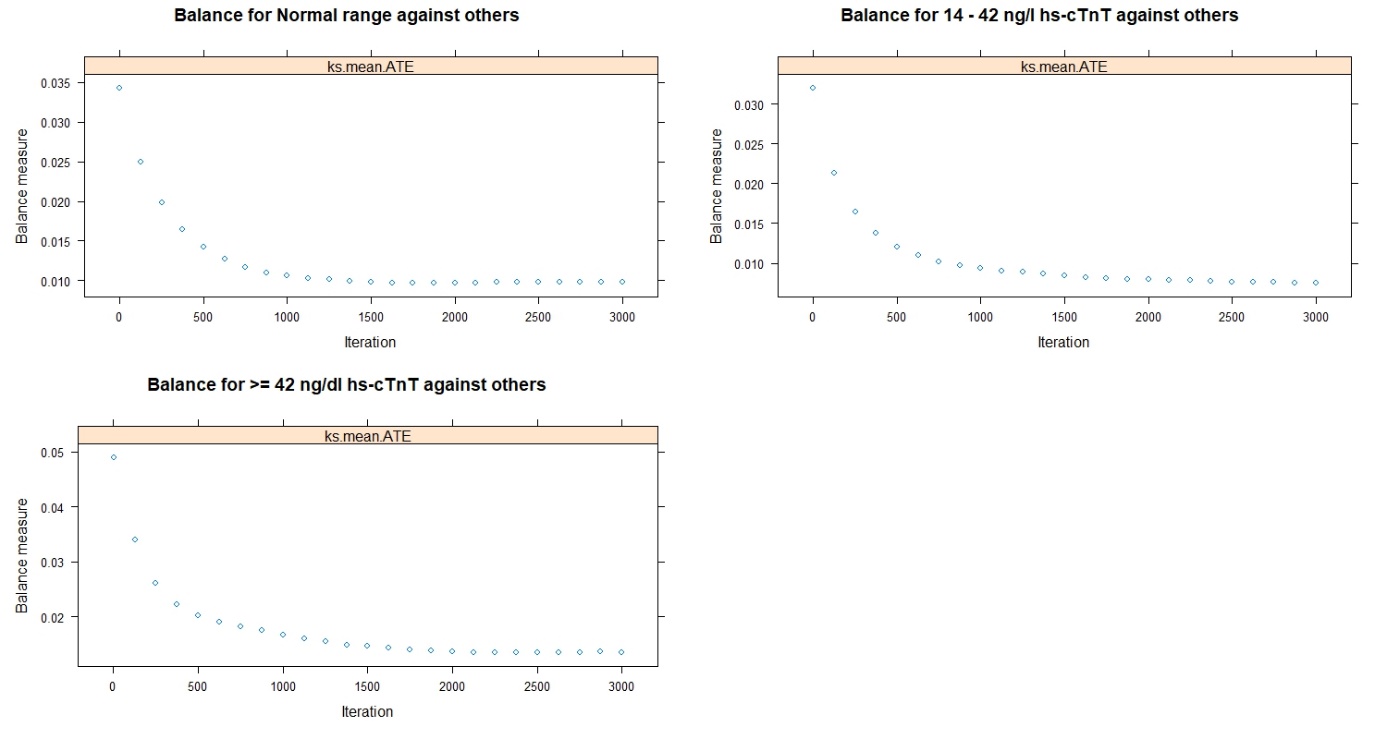


Estimated balance comparing each group to the pooled sample of the other groups over each iteration of the gradient boosted model used for propensity score estimation. ks: Kolmogorov-Smirnov; ATE: Average treatment effect.

**Supplemental Figure 2:** Propensity score weighing - absolute standardized mean difference


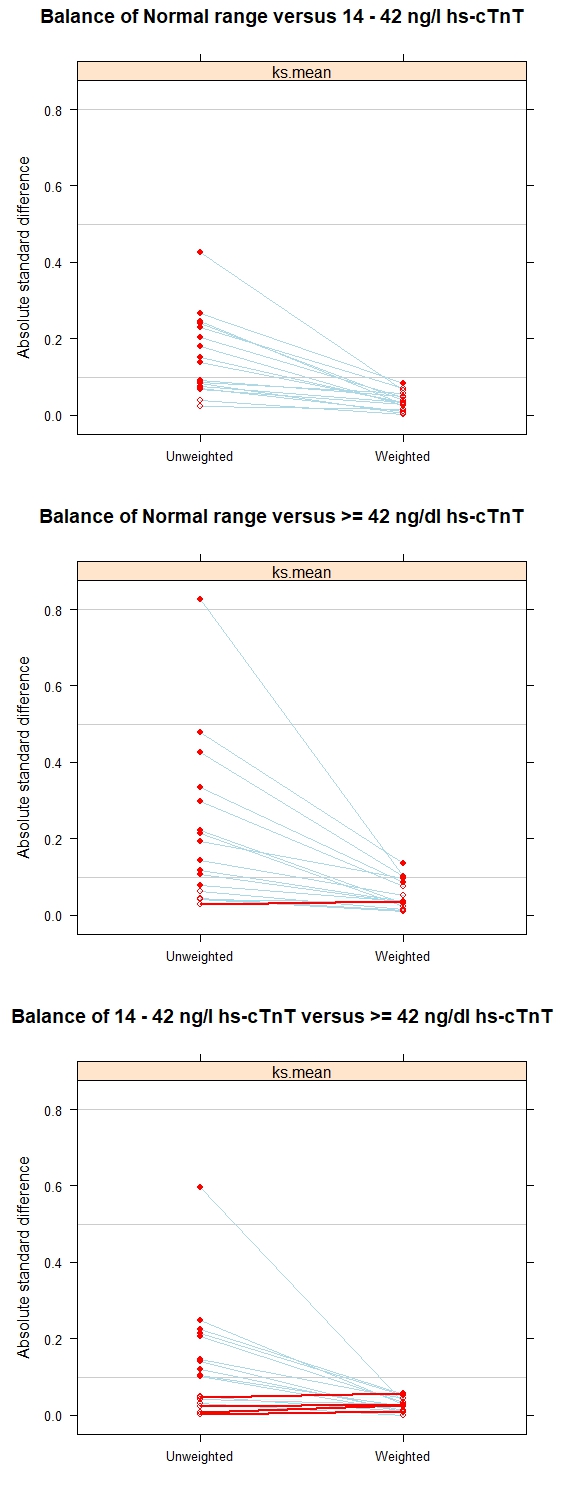


Absolute standardized mean difference (ASMD) comparing the balance of all covariates before and after weighing. Thick red lines denote increased ASMD of covariates after weighing. ks: Komogorov-Smirnov; ATE: Average treatment effect.

**Supplemental Figure 3:** Histogram of preoperative hs-cTnT


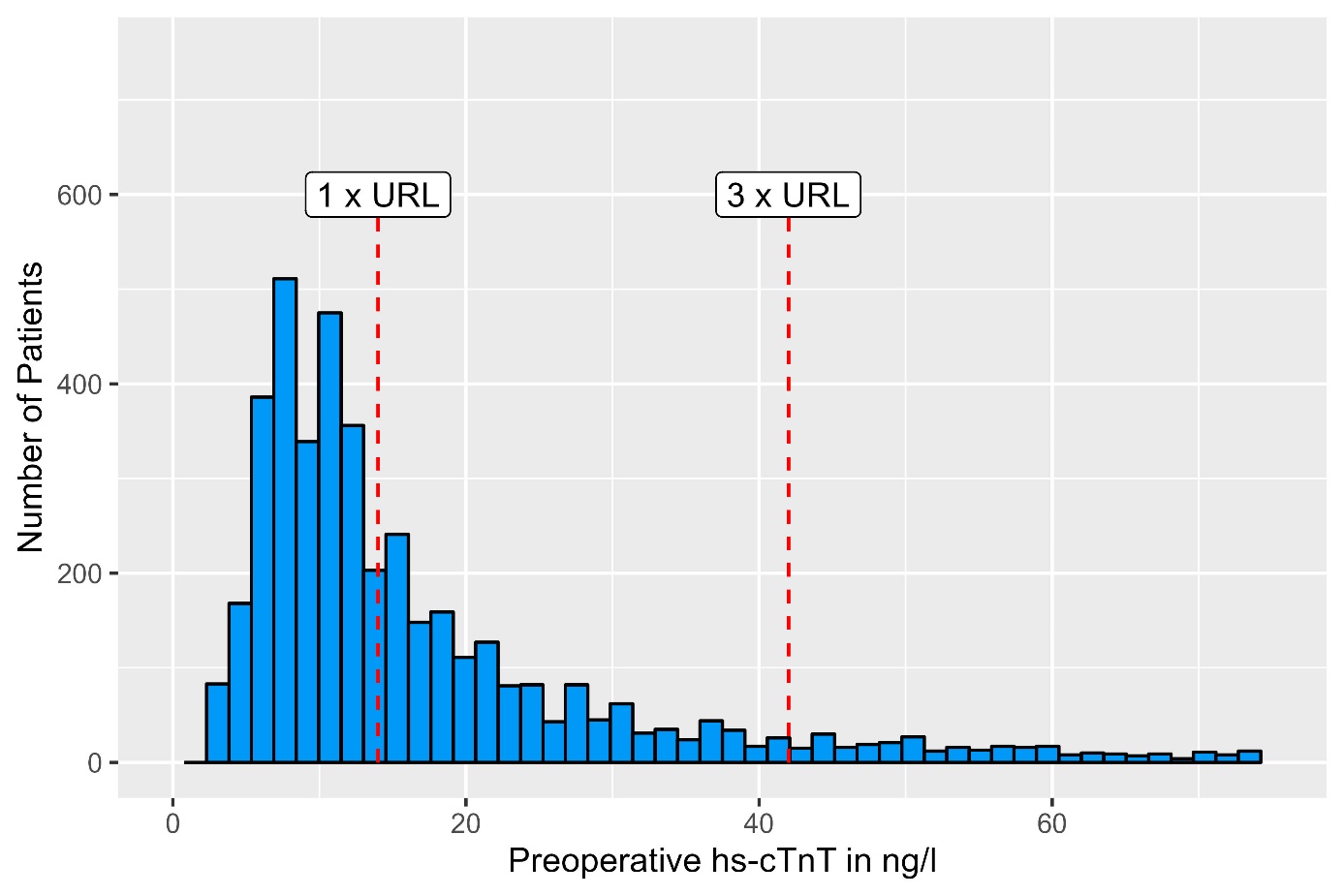


Hs-cTnT levels were measured the day before surgery. URL = 14ng/l; URL = URL = upper reference limit of normal.

**Supplemental Figure 4:** 30-day mortality is dependent on preoperative hs-cTnT


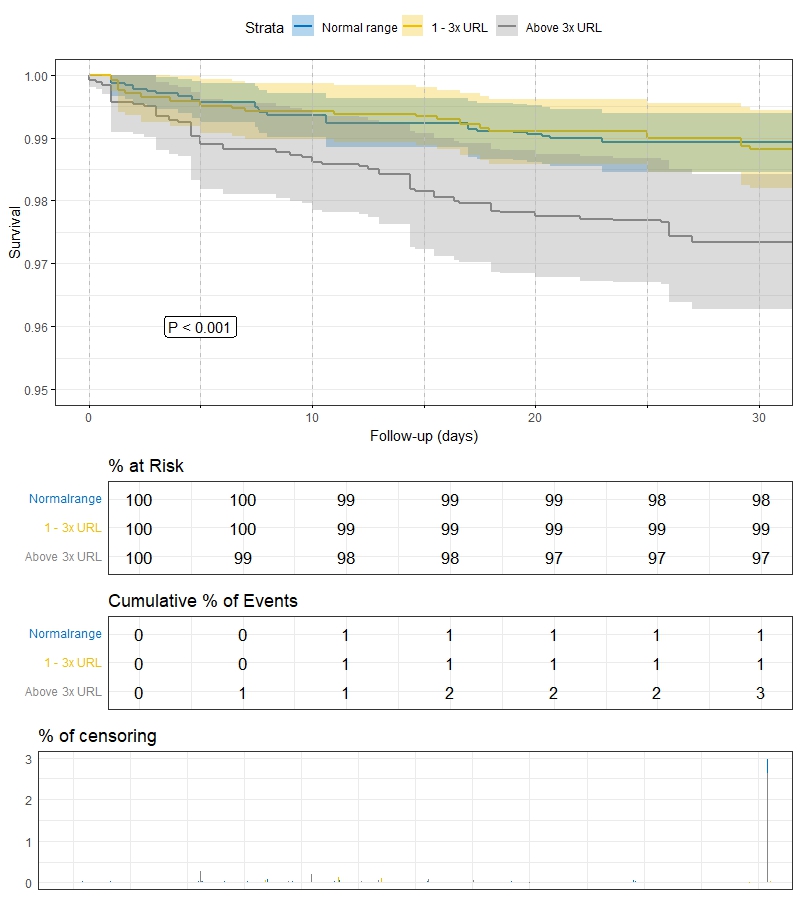


Patients were grouped based on their preoperative hs-cTnT values and a pseudo randomization using a propensity score weighing method was performed (<1x URL = blue; 1-3x URL = yellow; > 3xURL); URL = upper reference limit of normal.

**Supplemental Figure 5:** Preoperative hs-cTnT is an independent risk factor for the outcome after CABG


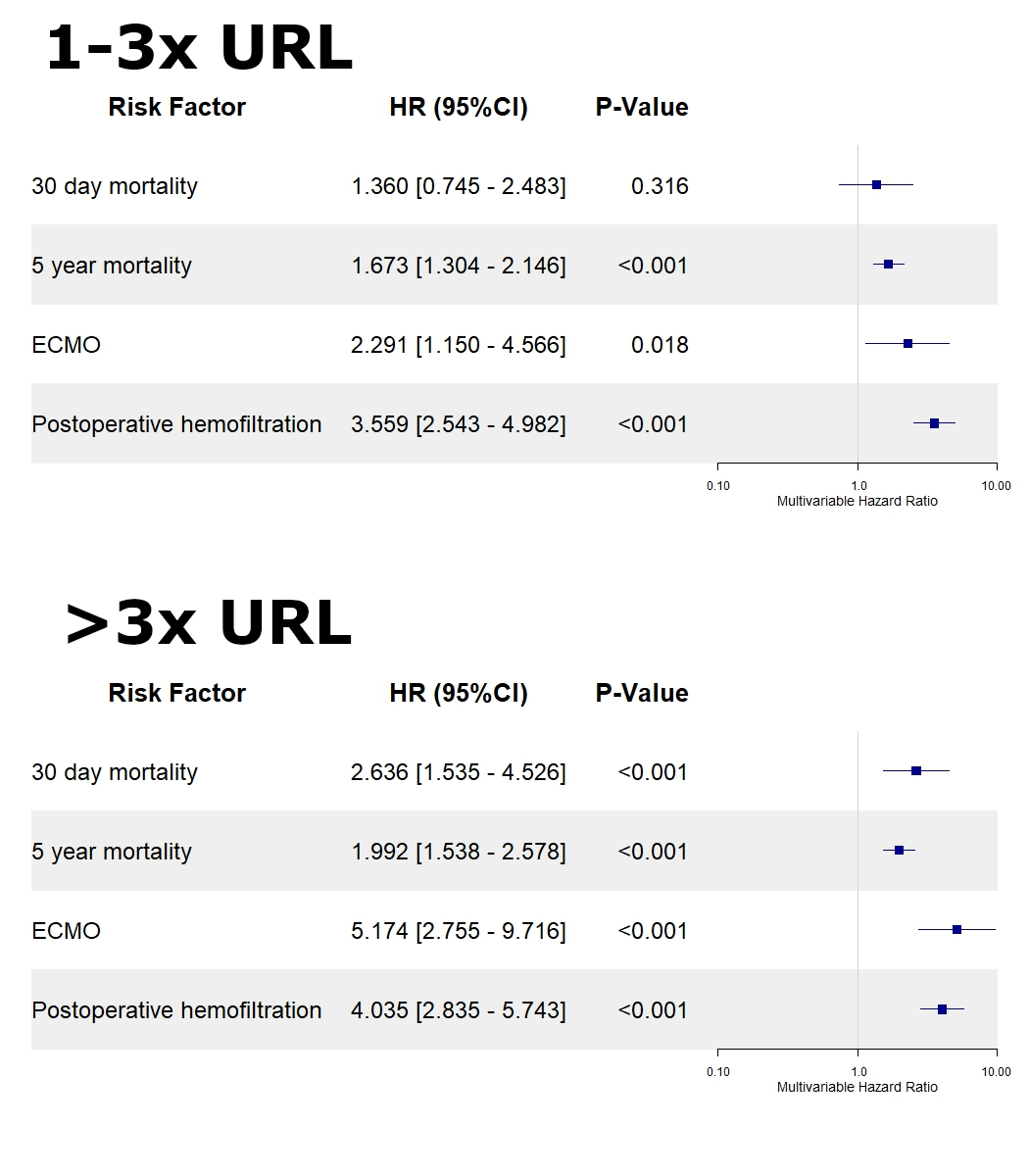
Patients were grouped based on their preoperative hs-cTnT values. Patients with preoperative hs-cTnT 1x – 3x URL, and in >3x URL were compared to <1x URL. Models were adjusted for the EuroSCORE II. CI = confidence interval; ECMO = Extracorporeal membrane oxygenation, URL = upper reference limit of normal.

**Supplemental Figure 6:** 5-year mortality is dependent on preoperative hs-cTnT


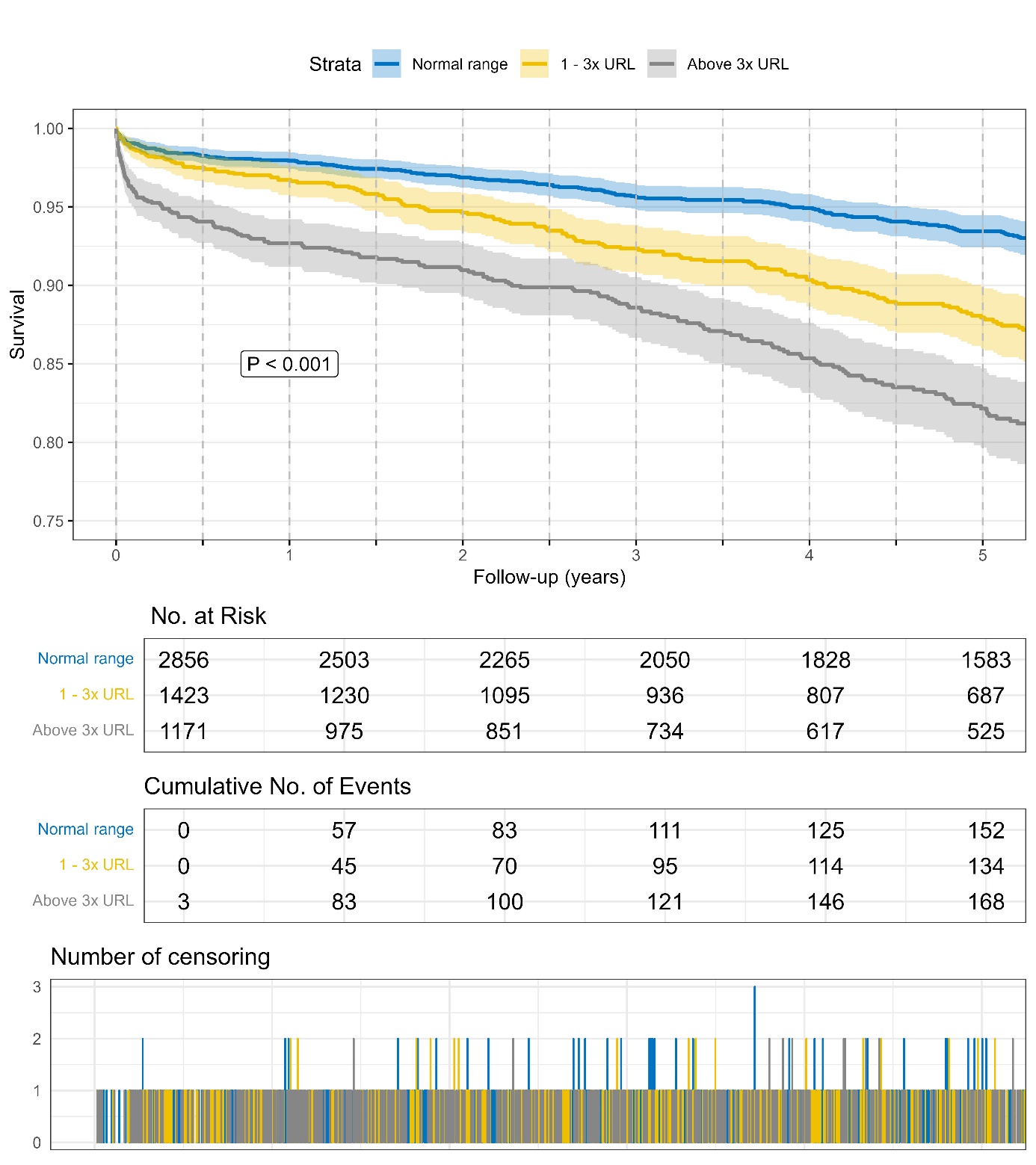


Patients were grouped based on their preoperative hs-cTnT values (<1x URL = blue; 1-3x URL = yellow; > 3xURL); URL = upper reference limit of normal.

**Supplemental Figure 7:** 30-day mortality is dependent on preoperative hs-cTnT


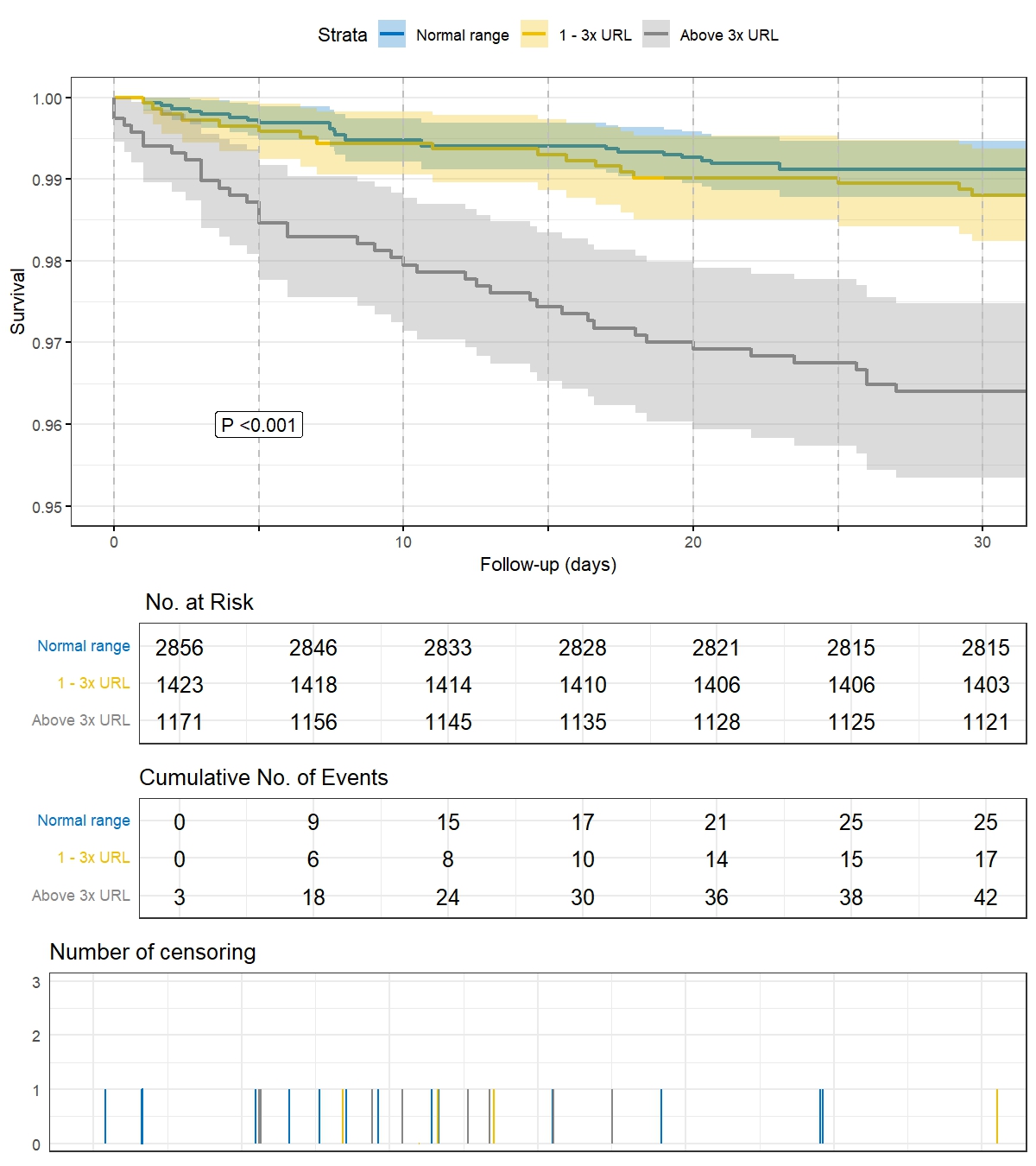


Patients were grouped based on their preoperative hs-cTnT values (<1x URL = blue; 1-3x URL = yellow; > 3xURL); URL = upper reference limit of normal.

**Supplemental Figure 8:** 30-day mortality is dependent on preoperative hs-cTnT in elective patients


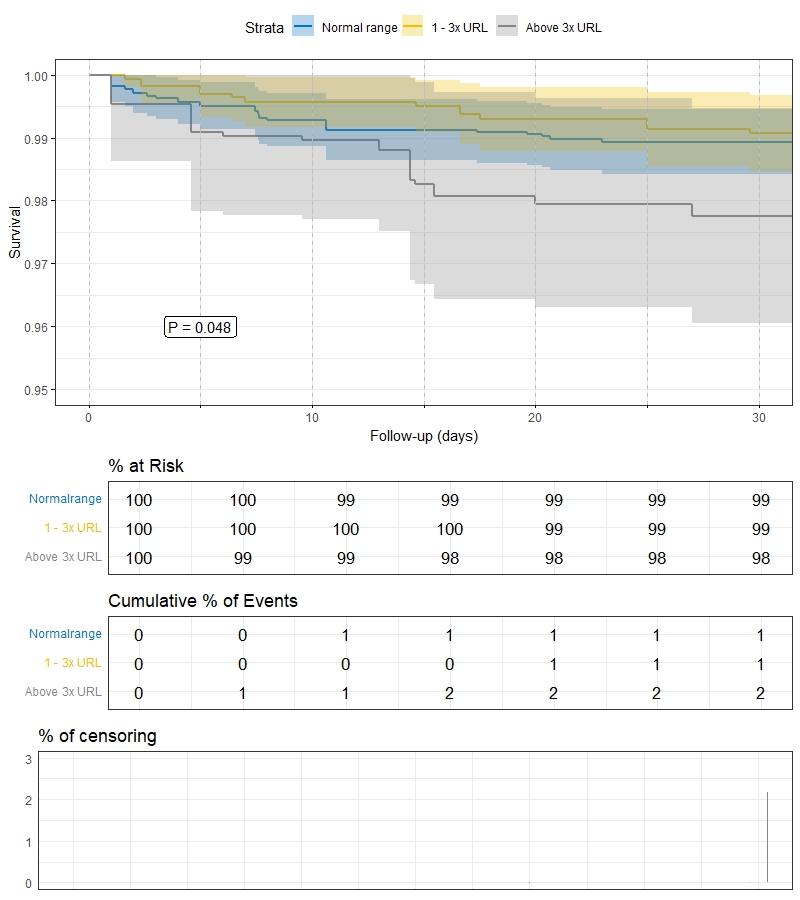


Only elective patients were analyzed and grouped based on their preoperative hs-cTnT values. A pseudo randomization using a propensity score weighing method was performed. (<1x URL = blue; 1-3x URL = yellow; > 3xURL); URL = upper reference limit of normal.

**Supplemental Figure 9:** Preoperative hs-cTnT is an independent risk factor for the outcome after elective CABG.


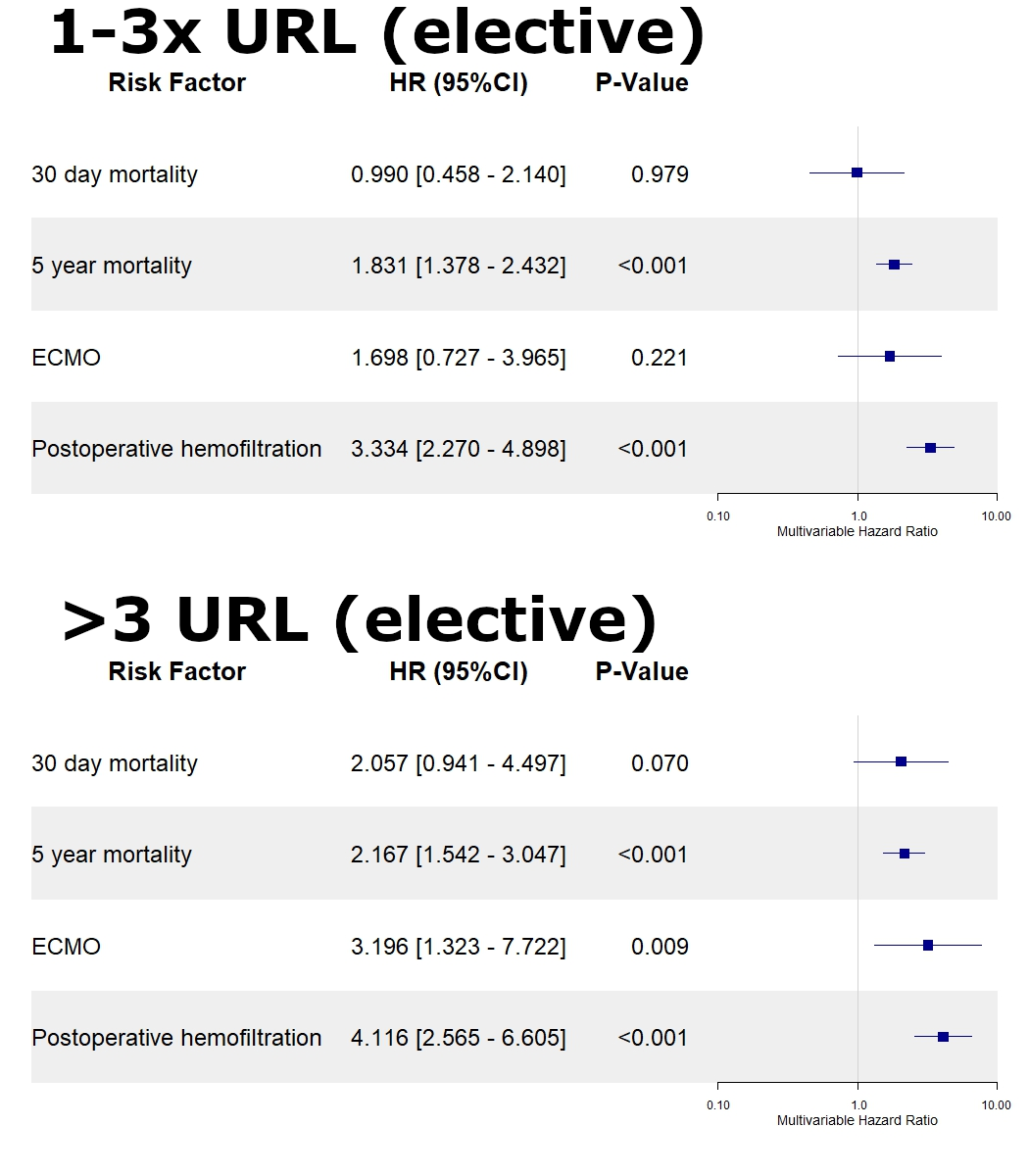


Only elective patients were analyzed and grouped based on their preoperative hs-cTnT values. Patients with preoperative hs-cTnT in 1x – 3x URL, and >3x URL were compared to <1x URL. Model was adjusted for the EuroSCORE II. CI = confidence interval; ECMO = Extracorporeal membrane oxygenation, URL = upper reference limit of normal.

**Supplemental Figure 10:** 5-year mortality is dependent on preoperative hs-cTnT in elective patients


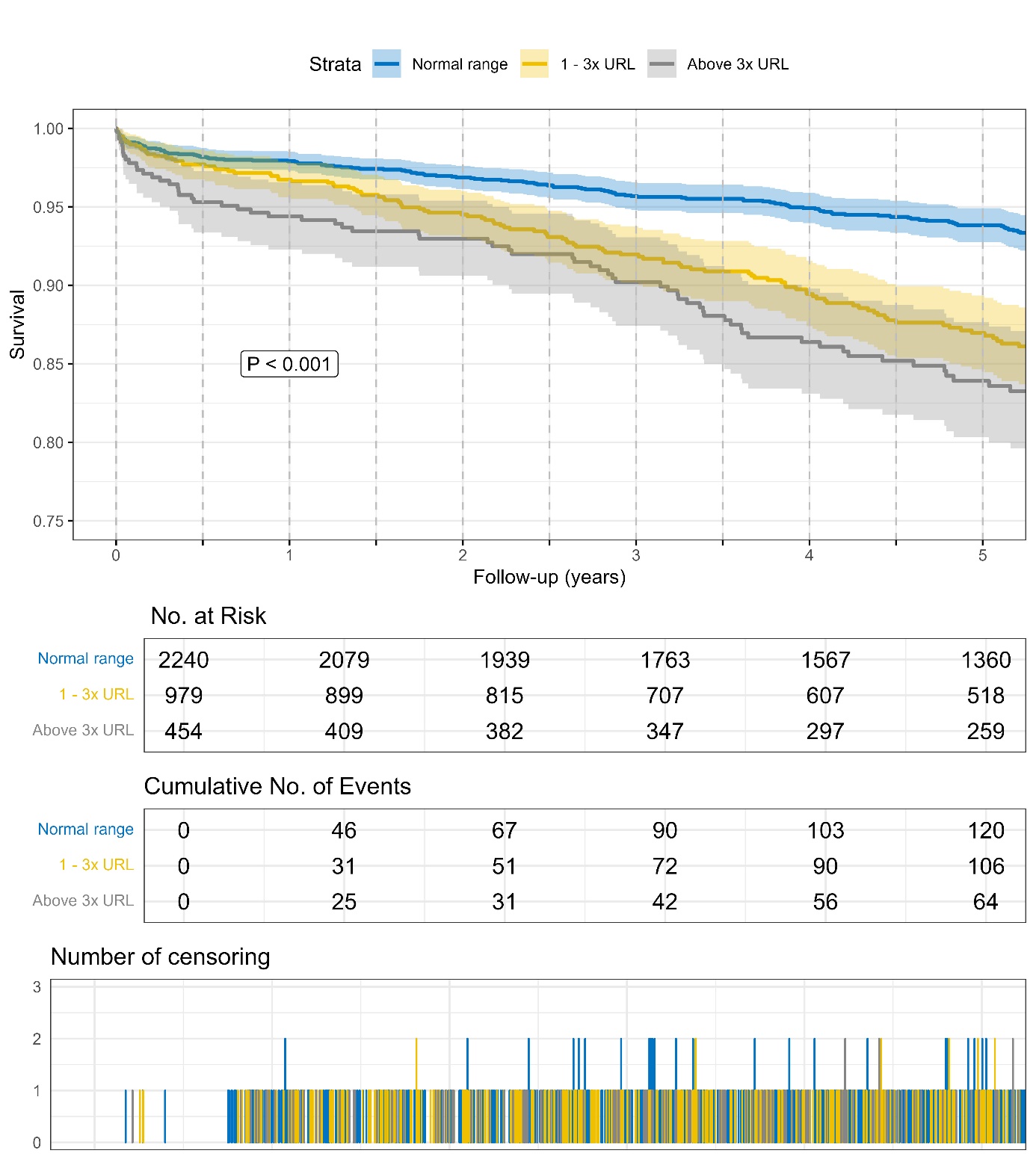


Only elective patients were analyzed and grouped based on their preoperative hs-cTnT values (<1x URL = blue; 1-3x URL = yellow; > 3xURL); URL = upper reference limit of normal.

**Supplemental Figure 11:** 30-day mortality is dependent on preoperative hs-cTnT in elective patients


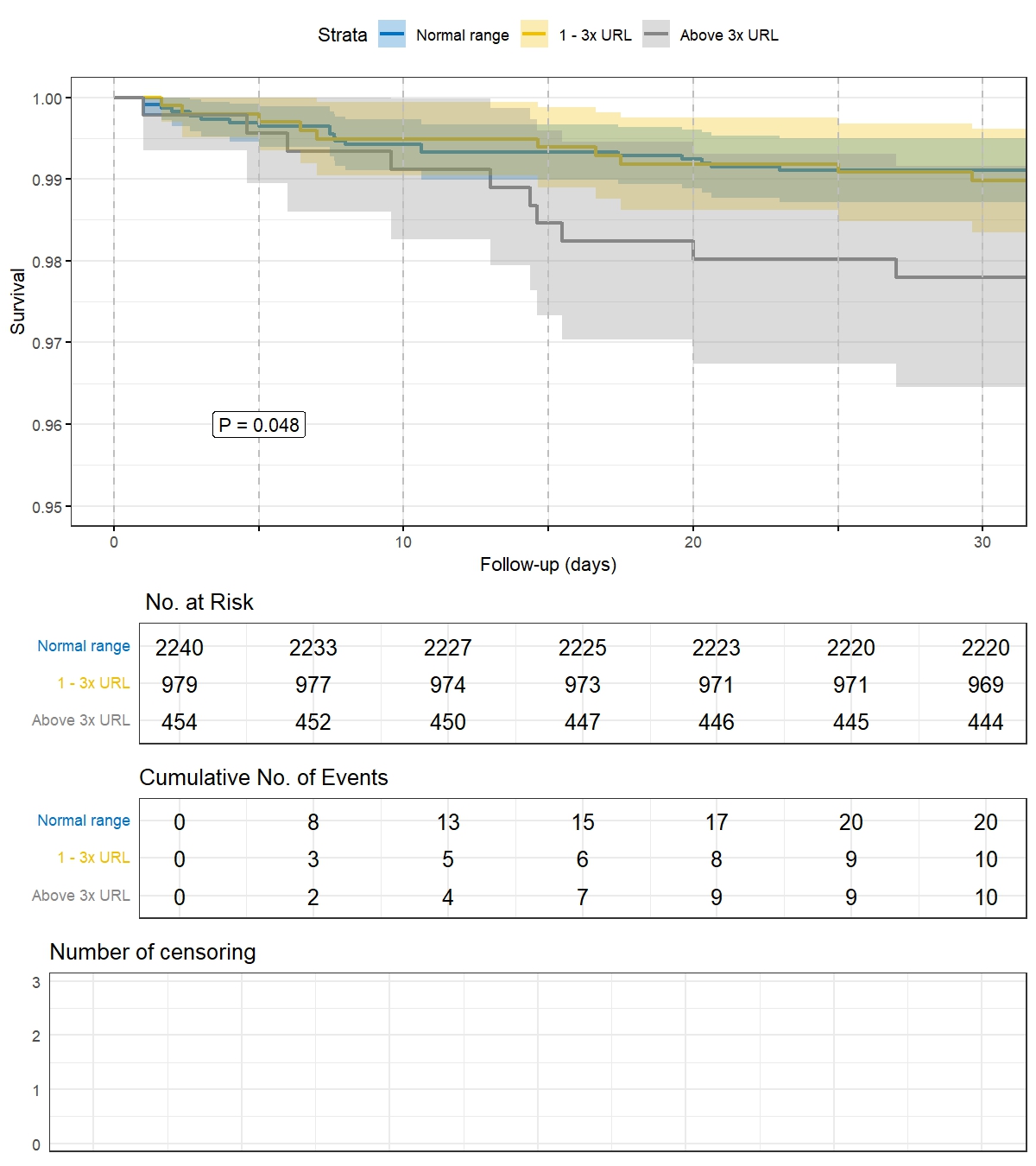


Only elective patients were analyzed and grouped based on their preoperative hs-cTnT values (<1x URL = blue; 1-3x URL = yellow; > 3xURL); URL = upper reference limit of normal.

**Supplemental Figure 12:** 5-year mortality in patients without chronic kidney disease depends on the preoperative hs-cTnT value.


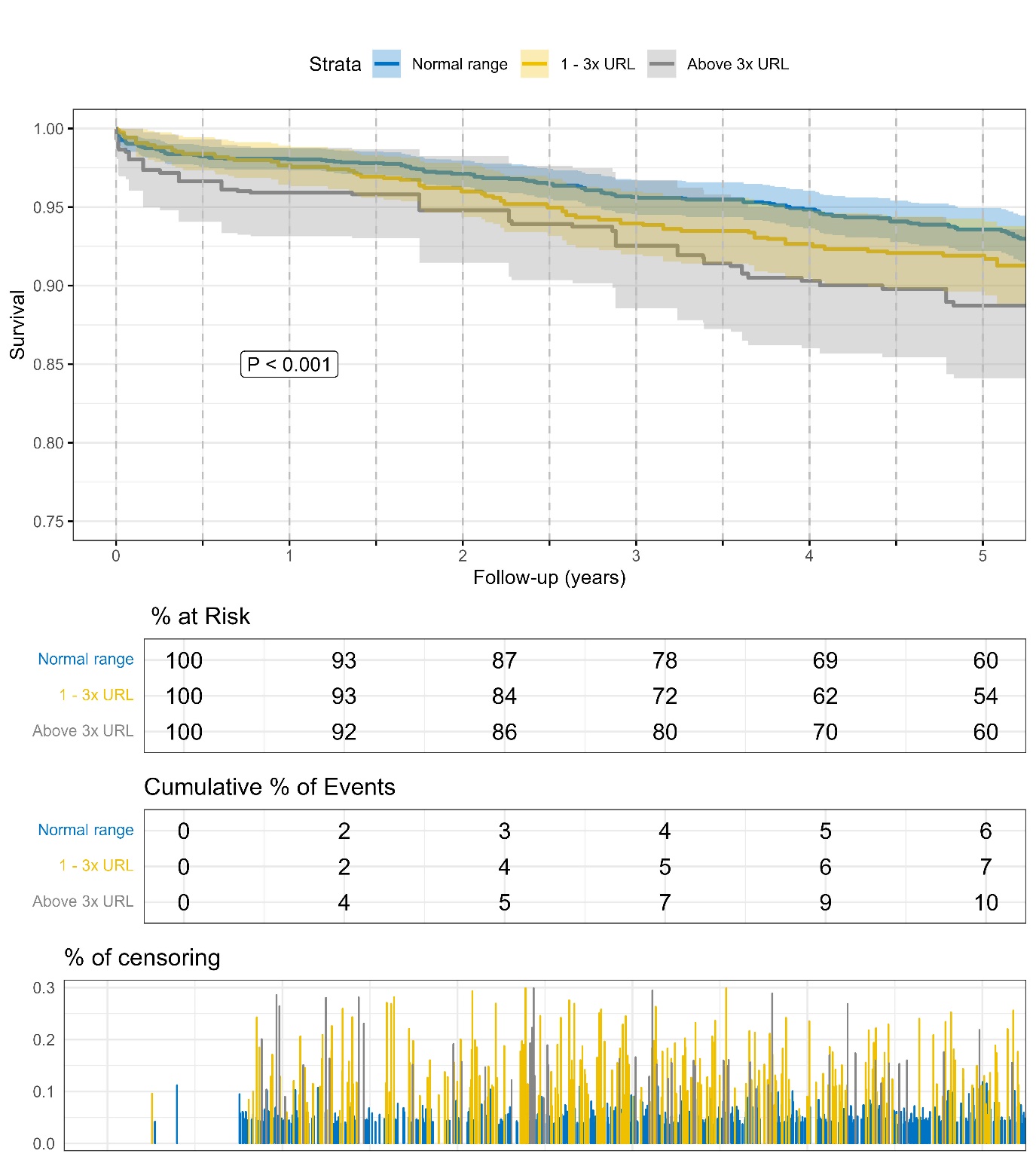


Only women with a creatine clearance <0.9 mg/dl and men with a creatine clearance < 1.2 mg/dl were analyzed and grouped based on their preoperative hs-cTnT values (<1x URL = blue; 1-3x URL = yellow; > 3xURL). A pseudo randomization using a propensity score weighing method was performed. URL = upper reference limit of normal.

**Supplemental Figure 13:** 5-year mortality in patients without chronic kidney disease depends on the preoperative hs-cTnT value.


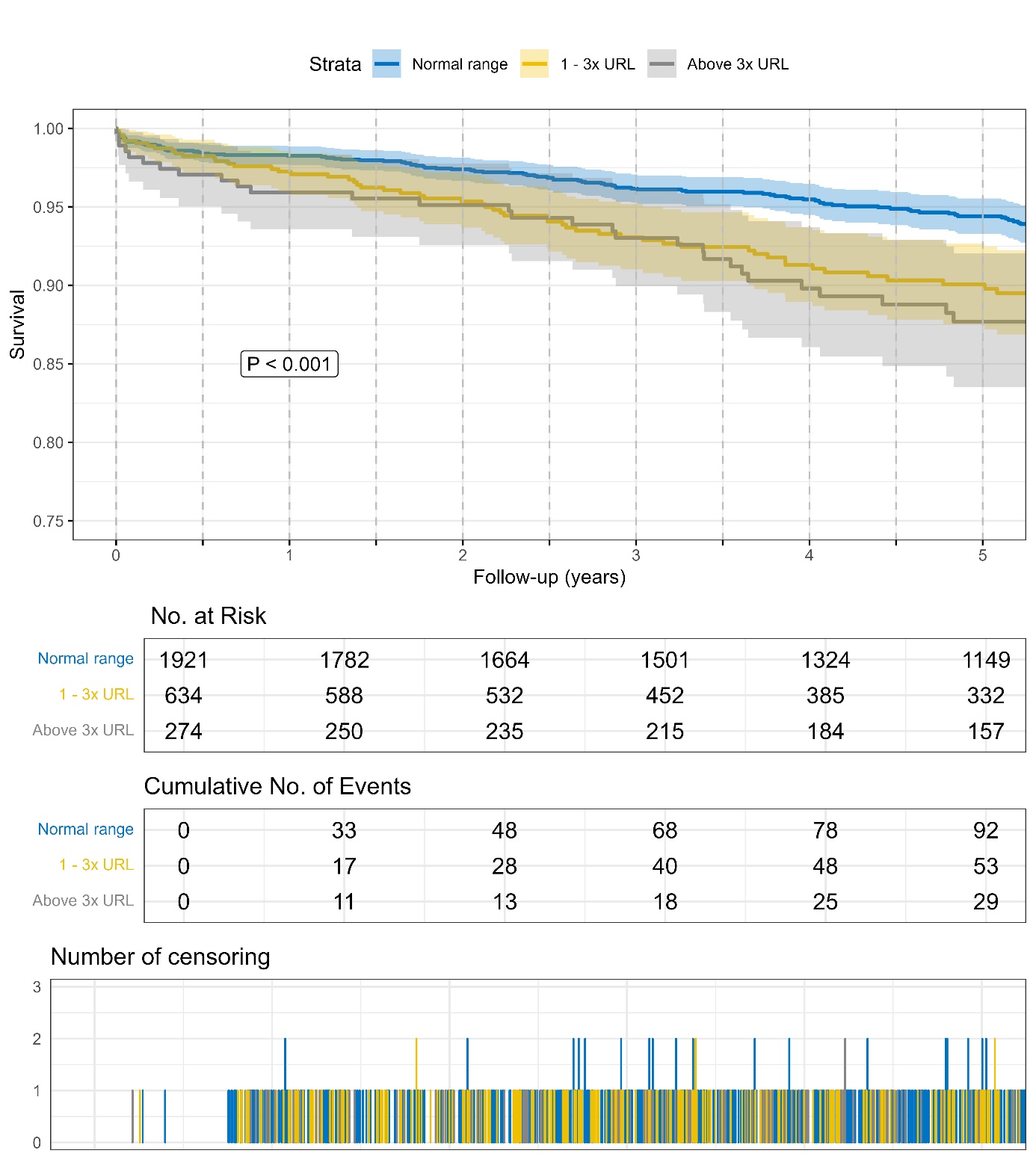


Only women with a creatine clearance <0.9 mg/dl and men with a creatine clearance < 1.2 mg/dl were analyzed and grouped based on their preoperative hs-cTnT values (<1x URL = blue; 1-3x URL = yellow; > 3xURL); URL = upper reference limit of normal.
